# Optical and surface energy probe of Hamaker constant in copper oxide thin films for NEMS and MEMS stiction control applications

**DOI:** 10.1038/s41598-021-83653-8

**Published:** 2021-02-19

**Authors:** Abraham Ogwu, T. H. Darma

**Affiliations:** 1 School of Engineering and Computing, University of the West of Scotland, Paisley Campus, PA1 2BE Scotland, UK; 2 Present Address: D. Serikbayev East Kazakhstan State Technical University, Ust-Kamenogorsk, Republic of Kazakhstan; 3 Present Address: Faculty of Science, Bayero University, Kano, Nigeria

**Keywords:** Materials science, Nanoscience and technology

## Abstract

Copper oxide films hold substantial promise as anti-stiction coatings in micro-electromechanical (MEMS) devices and with shrinking dimensions on the nanometre scale on nano electromechanical (NEMS) devices. The Hamaker constant will play a very significant role in understanding stiction and tribology in these devices. We used an approximate but sufficiently accurate form of the Lifshitz theory using the multiple oscillator model to calculate the Hamakers constant of symmetric copper oxide thin films based on experimentally obtained dielectric data in the wavelength range 190-850 nm using spectroscopic ellipsometry. We also used the Tabor–Winterton approximation (TWA) and Surface energy measurements to determine the Hamaker constant. There was better agreement in the Hamaker constant values obtained by the limited Lifshitz theory and TWA approach than with the Surface energy approach. The difference is explained through the influence of surface roughness on the surface energy using extensions of the stochastic KPZ growth model and the Family-Vicsek scaling relation and rigorous treatment of the Cassie-Baxter and Wenzel models as optimisations of a surface free energy functional linking roughness and surface tension. The dominance of the Cu_2_O phase in the films and of the London dispersion force on the surface of the films was previously confirmed by FTIR Cu(I)–O vibrational mode observation and XPS Cu 2*p*_3/2_ binding energy peak and its fitted satellites. The use of the limited Lifshitz theory and ellipsometry data would seem to provide a suitable best first approximation for determining the Hamaker constant of predominantly dispersive anti-stiction coatings in technologically important MEMS/NEMS devices.

## Introduction

The Van der Waals and Casmir forces, resulting from quantum mechanical dispersion and electrodynamic vacuum fluctuation, will play a very significant role in understanding stiction and tribology in future micro-electromechanical (MEMS) devices and with shrinking dimensions on the nanometre scale, on Nano-electromechanical (NEMS) devices. These devices will be of central importance as future Nano-sensors and Nano-actuators for both terrestrial and possible extra-terrestrial applications^1–4^. The significant surface to volume ratio in MEMS/NEMS devices, results in a pronounced manifestation of surface adhesion forces that will have contributions from capillary, electrostatic, chemical, Van der Waals and Casmir forces on the nanoscale. These adhesive forces will affect the expected performance of these devices. Many of the future MEMS and NEMS devices such as micro-mirrors, micro-resonators, nano-tweezers, nano-actuators etc., will operate in the electro-statically actuated mode. However, a major challenge with operating MEMS and NEMS devices in the electro-statically actuated mode is the electrostatic pull-in stability, limiting the possible applied electrostatic voltage, as well as the performance of the capacitance sensing system, when two surfaces stick together due to stiction^5–7^.

The pull-in phenomena (i.e. pull-in stability) is controlled by the pull-in parameters in electro-statically operated devices, namely the pull-in voltage and the pull-in gap, i.e. the voltage and gap distance limit of the switches The instability and buckling of MEMS and NEMS devices is controlled by stiction forces arising from Van der Waals and Casmir forces. This is usually tested by the electrostatic pull-in voltage/deflection set-up for electrostatic actuators^5–7^. The Casmir force between two uncharged surfaces is due to the modification of the zero-point energy associated with the electromagnetic modes between the two bodies and it is a geometry dependent force. The Van der Waals force on the other hand has both a geometry and material dependence. Palasantas and De Hosson^7^, also studied the effect of surface roughness on the Casmir Force between plates in MEMS/NEMS devices. They reported observing that the surface roughness exponent had a significant influence on the Casmir forces. The Casmir effect is known to operate at separations between two surfaces below 100 nm, whilst the Van der Waals force dominates at separations of the order of 10 nm and below^8,9^.

A lot of the investigations of the Casmir/Van der Waals mediated stiction forces in MEMS and NEMS devices in the literature, have concentrated on force measurements for different geometrical and electrostatic conditions using experimental and analytical modelling techniques^10^. This paper explores the material properties dependence of the Van der Waals force in thin films of Cu_2_O/CuO that can be used to fabricate MEMS/NEMS structures^11^. These films can also be used as anti-stiction coatings in both Ohmic and capacitive contact MEMs switches. Our approach is to use a physical probe technique based on ellipsometry and a chemical probe route through the extended Derjaguin–Landau–Verwey, Overbeek (XDLVO) surface energy approach to determine the Hamaker constant and its dependence on material properties. This will provide an additional route to selectively monitor and control the Van der Waals force contribution to the combined Casmir/Van der Waals forces operating on the surface of these and other thin films, when used in the fabrication of MEMS/NEMS structures, through a determination of the Hamaker constant, which is an indicator of the strength of the Van der Waals interaction.

## Theory

### Van der Waals force and its role on the growth and surface energy of materials surfaces

The total energy of a system of atoms and molecules consists of two major components, the bonded and non-bonded interaction energy terms. This is usually captured within the force field molecular mechanics modelling in the form,

1 $$ {\text{U}}_{{\text{total }}} = {\text{ U}}_{{{\text{bonded}}}} + {\text{ U}}_{{\text{non-bonded}}} $$

The bonded energy term is typically modelled using a Morse potential of the form,

2 $$ {\text{U }}(r_{ab} ) = {\text{D}}_{{\text{e }}} \left( {1 - e^{{ - \alpha \left( {r_{ab} {-} r_{ab,0} } \right)}} } \right) $$

D_e_ is the well-depth, α is a fitting parameter, r_ab_ is the separation between two atoms a and b in a two-body bonded interaction. The dominant non-bonded energy interaction term for uncharged surfaces is typically modelled with the Lennard–Jones potential for the Van der Waals interaction, expressed as,

3 $$ {\text{U}}_{LJ} = \varepsilon_{D} \left[ {\left( {\frac{\sigma }{r}} \right)^{12} - \, \left( {\frac{\sigma }{r}} \right)^{6} } \right] $$

є_D_ is a characteristic energy of dipolar interaction, σ is the distance of smallest approach and r is the separation distance. Attempts have already been made in the literature to show a correlation between the Morse and the Lennard–Jones potential, with the application of a modified form of the Morse potential used for describing non-bonded Van der Waals interactions^12,13^. The bonded and non-bonded energy of interactions in materials controls their growth, surface energy, wetting behaviour and surface roughness. Any detailed understanding and control of the surface energy of materials, such as the copper oxide thin films discussed in this paper for applications in nano-devices, has to involve the role of bonded interactions modelled through empirical potentials like the Morse potential and non-bonded interactions modelled through the Lennard–Jones potential for Van der Waals interactions. The present authors have reported ^14,15^, the important role of the Edwards and Wilkinson (E–W) equation of the form,

Edwards and Wilkinson (E–W) equation of the form,

4 $$ \frac{{\partial {\varvec{h}}}}{{\partial {\varvec{t}}}} = \, {\mathbf{v}}\nabla^{2} {\mathbf{h}} \, + {\varvec{\eta}}({\mathbf{r}}, \, {\mathbf{t}}) $$

In understanding the micro-structural development during thin film growth in a continuum model, where h(r, t), the average height of deposited particles on a surface is expressed as, h(r, t) = H(r, t) − <H> , H(r, t) is the height at a particular position (r) and time (t) and  <**H**>  is the mean height. $\nabla^{2}$ h is a diffusion process during growth and v is a surface tension term. ${\varvec{\eta}}$ (r, t) is a Gaussian noise term with a zero mean and lacks spatial or temporal correlation.

This was further extended in our analysis to include contributions to lateral growth of the thin films through the Kardar, Parisi and Zhang (KPZ) equation, expressed as^16^,

5 $$ \frac{{\partial {\varvec{h}}}}{{\partial {\varvec{t}}}} = \, {\mathbf{v}}\nabla^{2} {\mathbf{h}} \, + \frac{{\varvec{\lambda}}}{2}\left( {\nabla {\varvec{h}}} \right)^{2} + {\varvec{\eta}}\left( {{\mathbf{r}}, \, {\mathbf{t}}} \right) $$

The term $\frac{{\varvec{\lambda}}}{2}$ $\left( {\nabla {\varvec{h}}} \right)^{2}$ accounts for the presence of lateral growth with a coefficient **λ**. The KPZ and E-W equations defy analytical solutions except for the simplest cases in one dimension. Watson et al.^17^ have proposed an explicit model relating surface tension (γ) to the variation in thickness h (x) during a template growth of a nanowire, based on a 1 + 1-dimension computer simulation. They proposed an equation of the form,

6 $$ \frac{{ - \partial {\mathbf{G}}}}{{\partial {\mathbf{h}}}} = \frac{{\partial {\varvec{h}}}}{{\partial {\varvec{t}}}} = \, {\mathbf{v}}\nabla^{2} {\mathbf{h}} \, + {\varvec{\eta}}\left( {{\mathbf{r}}, \, {\mathbf{t}}} \right) $$

For growth under local equilibrium and small gradients, where,

7 $$ {\mathbf{G}} \, = \, {\mathbf{2}} \, {\mathbf{\pi \gamma }}\smallint \sqrt {1 + {\varvec{h}}^{2} } {\mathbf{dx}} $$

This is a functional form of the Edwards-Wilkinson equation for film growth, with G as the Surface free energy expressed in a functional form. Watson et al. further tried to model the free energy of their model system and that of a smooth nanowire and obtained an equation similar to the KPZ equation^18^. They suggested based on numerical simulation and scaling arguments, that scaling exponents for the formation of nanowires by templating reactions, showed better agreement with the Edwards–Wilkinson model. This might not be un-connected to the approximating assumptions made in their analysis.

The significance of the Morse type potential is evident in the equilibrium wetting model expressed by the Lipowsky or Edwards–Wilkinson equation with a wall or potential of the form^18,19^,

8 $$ \frac{{\partial {\varvec{h}}}}{{\partial {\varvec{t}}}} = \, {\mathbf{v}}\nabla^{2} {\mathbf{h}} - \frac{{\partial {\varvec{V}}}}{{\partial {\varvec{h}}}} + {\varvec{\eta}}\left( {{\mathbf{r}}, \, {\mathbf{t}}} \right) $$

where V (h) is a Morse potential of the form,

9 $$ {\mathbf{V}} \, \left( {\mathbf{h}} \right) \, = \, {\mathbf{b}} \, \left( {\mathbf{T}} \right){\varvec{e}}^{{ - {\varvec{h}}}} + \, {\mathbf{C}}e^{ - 2h} $$

**T** is the temperature and **b (T)** vanishes linearly at the wetting temperature.

Under non-equilibrium wetting, Eq. (9) becomes a KPZ equation with a potential of the form,

10a $$ \frac{{\partial {\varvec{h}}}}{{\partial {\varvec{t}}}} = \, {\mathbf{v}}\nabla^{2} {\mathbf{h}} \, - \frac{{\partial {\varvec{V}}}}{{\partial {\varvec{h}}}} + \frac{{\varvec{\lambda}}}{2}\left( {\nabla {\varvec{h}}} \right)^{2} + {\varvec{\eta}}\left( {{\mathbf{r}}, \, {\mathbf{t}}} \right) $$

In the potential in the above continuum model of thin film growth, a Morse potential controls the surface tension (**γ**) at the interface between two phases **α/β** in direct contact, whilst the interaction potential between separated surfaces usually due to Van der Waals forces, modelled with a Lennard–Jones potential for uncharged surfaces, which contributes to the surface tension on the exposed surface of a material. The effect of the roughness on the surface energy comes from the height h(x, t) terms in the above equations. A Family–Vicsek scaling relation of the surface roughness in the KPZ equation is defined by the equation,

10b $$ W\left( {L,t} \right) = L^{\alpha } f\left( {\frac{t}{{L^{z} }}} \right) $$

where $\left( {L,t} \right)$, is a width function dependent on h(x, t) and α is a scaling function^11^.

This effect will now be explored to monitor the effect of varying the reactive magnetron sputtering deposition conditions for copper oxide thin films on Hamaker constant changes, monitored with the extended Derjaguin–Landau–Verwey–Overbeek (XDLVO) surface energy measurements.

### Contact angle and surface energy measurement including roughness contributions.

Contact angle measurement is used to relate the key thermodynamic parameters of a surface through the Young equation given by

11a $$ \gamma_{lv} \cos \theta = { }\gamma_{sv} - { }\gamma_{sl} $$

where γ_lv_, γ_sv_, and γ_sl_ are the liquid–vapour, solid–vapour, and solid–liquid interface energies respectively. The surface energy components provide necessary information for understanding the factors that underpin the durability and resistance of the films to environmental changes like water and fog condensation. The first two terms in Young’s equation can be determined experimentally, while the remaining terms are estimated based on theoretical considerations classified into either microscopic or macroscopic approaches for determining the surface energy. In the microscopic theories, the total surface energy can be expressed as

11b $$ \gamma = { }\gamma^{p} + { }\gamma^{d} $$

where γ^p^ is the polar component of the surface energy and γ^d^ is the dispersive component of the surface energy. The microscopic theories of surface energy can also be separated into two approaches depending on the type of combining rule used to determine the surface energy from its dispersive and polar components. These approaches are the geometric mean proposed by Fowkes, and Owens and Wendt. The second approach is the Lifshitz–van der Waals acid–base approach developed by Van Oss et al. Detail theory of these approaches are given in previous reports^11^. The effect of roughness on the contact angle and hence the surface energy is captured within the Wenzel and the Cassie-Baxter equations. In the case of a heterogeneous hydrophobic surface, the Cassie –Baxter equation takes the form given below^20^,

12a $$ \cos \vartheta_{A} = f_{1} \cos \vartheta_{1} + f_{2} \cos \vartheta_{2} $$

where *f*_1_ and *f*_2_ are area fractions of materials are phases and $\vartheta_{1 } and \vartheta_{2}$ are the contact angles on phases 1 and 2 respectively. The equation becomes,

12b $$ \cos \vartheta_{A} = f_{1} \cos \vartheta_{1} - f_{2} $$

If the entrapped phase on the surface is air.

A more general form of the Cassie-Baxter equation takes the form,

12c $$ \cos \vartheta^{*} = r_{f } f\cos \vartheta + f - 1 $$

$r_{f }$ is the roughness ratio, $f_{i}$ is the fraction of the total surface area, $\vartheta^{*}$ is the apparent contact angle, $\Theta$ is the Young contact angle.

In the case of a hydrophilic surface, the Wenzel equation is expressed as

12d $$ \cos \vartheta^{*} = r \cos \vartheta $$

$r$ is the roughness ratio.

The Cassie–Baxter equation contains the surface tension term through the relationship,

12e $$ \gamma \cos \vartheta^{*} = \mathop \sum \limits_{n = 1}^{N} f_{i} \left( {\gamma_{sv} - \gamma_{sl} } \right) $$

$\gamma_{sv}$ is the solid/vapour surface tension.

$\gamma_{sl}$ is the solid liquid surface tension.

### Reflectance ellipsometry

The complex refractive index N represents the real and imaginary parts of the refractive index:

13 $$ N = n + ik $$

where n is the refractive index and k is the damping constant (also known as the extinction coefficient) which describe a change in the phase velocity and amplitude, respectively, of the electromagnetic wave propagating through a medium.

The optical response is completely described by the complex dielectric response

14 $$ \varepsilon \left( {\uplambda } \right) = \varepsilon_{1} \left( {\uplambda } \right) + i\varepsilon_{2} \left( {\uplambda } \right) $$

This quantity is related to the complex refractive index N by the expression ε = N^2^. The real and imaginary parts of the dielectric response are therefore given by the expressions

15 $$ \varepsilon_{1} \left( {\uplambda } \right) = n^{2} \left( {\uplambda } \right) - k^{2} \left( {\uplambda } \right){\text{ and }}\varepsilon_{2} \left( {\uplambda } \right) = 2n\left( {\uplambda } \right)k\left( {\uplambda } \right) $$

The above relationship between the optical constants is investigated in our current work on copper oxide thin films using spectroscopic ellipsometry, to obtain dielectric data on our prepared copper oxide films in the wavelength range 190–850 nm, reported in this paper. This effect will now be explored to monitor the effect of varying the reactive magnetron sputtering deposition conditions for copper oxide thin films on Hamaker constant changes, monitored with spectroscopic ellipsometry.

### Hamaker constant

The surface forces between materials play an important role in their adhesion, wetting, stiction and other tribological phenomenon^21^. An important interaction between surfaces which is always present in materials on the nanoscale is the van der Waals interaction. The Hamaker constant represents a convenient way of estimating the magnitude of the van der Waals interaction between surfaces. It is obvious that an accurate determination of the Hamaker constant is necessary for an understanding of the effect of interfacial forces on the various phenomena mentioned above. For most solids and liquids, the Hamaker constant lies in the range 0.4–4 × 10^−19^ J^21^.

Rigorous methods for calculating the van der Waals force of interaction between two macroscopic bodies with an intervening medium between them was has been developed by a number of researchers in the literature^22–24^, based on the fluctuations in the electromagnetic field between two macroscopic bodies, modified by the separating medium. The Hamaker constant was estimated from the frequency dependent dielectric properties of the components and is expressed by the equation^22–24^.

16 $$ A_{132} = - \frac{3kT}{2} \sum \limits_{m}^{\infty }{^\prime }  \sum \limits_{s = 1}^{\infty } \frac{{\left( {\Delta_{13} \Delta_{23} } \right)^{s} }}{{s^{3} }} $$

where ${\Delta }_{kl} = \frac{{\varepsilon_{k} \left( {i\xi_{n} } \right) - \varepsilon_{l} \left( {i\xi_{n} } \right)}}{{\varepsilon_{k} \left( {i\xi_{n} } \right) + \varepsilon_{l} \left( {i\xi_{n} } \right)}}$, and $\xi_{n} = n\frac{{\left( {4\pi^{2} kT} \right)}}{h}$, h is Plank’s constant, and n = (0,1,2….).

For non polar materials,

17 $$ \varepsilon \left( {i\xi } \right) = 1 + \frac{{C_{IR} }}{{1 + \left( {{\raise0.7ex\hbox{$\xi $} \!\mathord{\left/ {\vphantom {\xi {\omega_{IR} }}}\right.\kern-\nulldelimiterspace} \!\lower0.7ex\hbox{${\omega_{IR} }$}}} \right)^{2} }} + \frac{{C_{UV} }}{{1 + \left( {{\raise0.7ex\hbox{$\xi $} \!\mathord{\left/ {\vphantom {\xi {\omega_{UV} }}}\right.\kern-\nulldelimiterspace} \!\lower0.7ex\hbox{${\omega_{UV} }$}}} \right)^{2} }} $$

C_IR_ and ω_IR_, and C_UV_ ω_UV_ are the function oscillator strengths and absorption frequencies in the infrared and ultraviolet regions respectively. The static permittivity ε(0) is represented as

18 $$ \varepsilon \left( 0 \right) = 1 + \mathop \sum \limits_{1 = 1}^{N} C_{i} $$

where $$C_{i} = \frac{2}{\pi }\frac{{f_{i} }}{{\omega_{i} }}$$.

C_IR_ is roughly represented as

19 $$ {\text{C}}_{{{\text{IR}}}} = \, \omega \left( 0 \right) \, {-}{\text{ C}}_{{{\text{UV}}}} {-}{ 1} $$

The two parameters that characterise absorption spectra in the ultraviolet region C_UV_ and ω_UV_ are obtained from Hough and White relation and represented as^23,24^;

20 $$ n^{2} - 1 = \left( {n^{2} - 1} \right)\frac{{\omega^{2} }}{{\omega_{UV}^{2} }} + C_{UV} $$

In the case of transparent substances in the visible light range, a linear Cauchy plot is performed with the vertical axis of (n^2^ – 1) and the horizontal axis of (n^2^ – 1)ω^2^, (ω_UV_)^−2^ is obtained by its gradient, and C_UV_ is obtained by its y intercept^23,24^.

In the absence of the absorption frequencies of the three media, a simpler approach is to assume the frequencies to be the same leading to the Tabor-Winterton approach of evaluating the Hamaker constant as^24^;

21 $$ A_{132} \approx \frac{3}{4} kT\left( {\frac{{\varepsilon_{1} - \varepsilon_{3} }}{{\varepsilon_{1} + \varepsilon_{3} }}} \right)\left( {\frac{{\varepsilon_{2} - \varepsilon_{3} }}{{\varepsilon_{2} + \varepsilon_{3} }}} \right) + \frac{{3h\nu_{e} }}{8\sqrt 2 }\frac{{\left( {n_{1}^{2} - n_{3}^{2} } \right)\left( {n_{2}^{2} - n_{3}^{2} } \right)}}{{\left( {n_{1}^{2} + n_{3}^{2} } \right)^{1/2} \left( {n_{2}^{2} + n_{3}^{2} } \right)^{1/2} \left\{ {\left( {\left( {n_{1}^{2} + n_{3}^{2} } \right)^{1/2} + \left( {n_{2}^{2} + n_{3}^{2} } \right)^{1/2} } \right)} \right\}}} $$

where k is Boltzmann constant, T is temperature, and $\nu_{e}$ is the plasma frequency of the free electron gas.

The Hamaker constant A_H_ can also be evaluated from knowledge of the surface energy and vice versa according to the relation^22^;

22 $$ \gamma_{s} = \frac{{A_{H} }}{{24\pi D_{o}^{2} }} $$

where γ_s_ is the surface energy (mJ/m^2^), D_o_ is the cut-off distance (~ 0.165 nm).

Another simpler method of evaluating the Hamaker constant is given as^20^;

23 $$ A_{H} = \frac{3kT}{2} \mathop \sum \limits_{{\begin{array}{*{20}c} {sampling} \\ { frequency} \\ \end{array} }} \left( {\frac{{\varepsilon_{1} - \varepsilon_{3} }}{{\varepsilon_{1} + \varepsilon_{3} }} .\frac{{\varepsilon_{2} - \varepsilon_{3} }}{{\varepsilon_{2} + \varepsilon_{3} }}} \right).Rel\left( l \right) $$

The function $Rel\left( l \right)$ is the relativistic retardation function which suppresses interactions due to the finite velocity of light. It is important for sampling frequencies that are of the order of the travel time of the atom interactions.

## Experimental investigation

### Thin film deposition

The film deposition was performed with a cryo-pumped vacuum chamber (CVC) rf magnetron sputtering unit AST304 located at the University of the West of Scotland, U.K. The deposition chamber is 25″ in diameter with a target-substrate separation of 9.5 cm. The materials are solid copper target of 99.99% purity, 8.0″ diameter and 6.0 mm thick Prior to deposition, the chamber was evacuated to a base pressure of 10^–6^ Torr. Glass slides, silica and silicon wafer which were cleaned ultrasonically with iso-propanol and then washed with de-ionised water were used as substrates. High purity argon and oxygen were used as the sputtering and reactive gases respectively. The target was pre-sputtered in pure Argon atmosphere for 3 min to remove oxide layers if any on the surface of the target. All depositions are for duration of five (5) minutes. The deposition conditions are given in Table 1.

**Table 1:** Film deposition condition.

Power (W)	Power density (W/cm^2^)	Pressure (mTorr)	Oxygen flow range (sccm)
200	0.6	2.0–6.0	1–4
300	0.9	2.0–6.0	1–4
400	1.2	2.0–6.0	2–6
500	1.5	2.0–6.0	2–6
600	1.9	2.0–6.0	2–6

### Film characterisation

**Figure 1:**
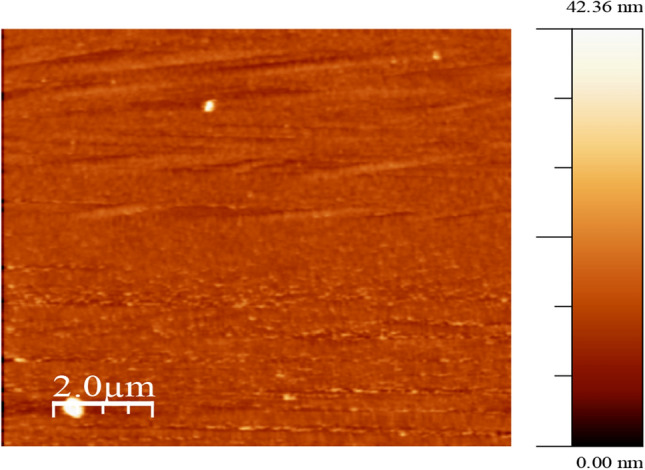
AFM topography of film prepared at 300 W power, 3sccm oxygen flow and 6.0mTorr pressure.

The film characterisation was conducted with the following equipment located at the University of the West of Scotland; UK. X-Ray diffraction (XRD) patterns of the prepared samples were recorded on a Siemens D5000 X-ray Diffractometer using CuKα radiation- to identify the copper oxide phases present and their crystal structures in the films prepared on microscope glass slides and silica substrates. The XRD spectra in previously reported in ref.^14^ showed that the films prepared at different deposition conditions are predominantly of the Cu_2_O phase containing crystallites in a predominantly amorphous structure. This has been previously reported by the present authors^14^. The average crystallite sizes were found to be between 46 and 65 nm. A Hitachi S-4100 scanning electron microscope (SEM) with a Germanium detector (Oxford Instruments) was used to obtain surface micrographs, cross-section, and elemental composition of films prepared on microscope glass substrates. A thin gold coating was applied on the film surfaces to enhance the cross-sectional image and carbon coating to enhance the surface imaging. The films prepared at forward power of 200 W–600 W with different combinations of oxygen flow rate and deposition pressure have dense columnar structures, some with rough surfaces have been reported previously by the present authors^14^. The surface roughness was seen to increase with increasing power. The topographical features and surface roughness of the films prepared on microscope glass substrates were investigated by atomic force microscope (AFM) imaging on a Digital Instruments Veeco Metrology system with a Si_3_N_4_ cantilever. The Nanoscope digital instrument AFM incorporates a roughness command which generates a wide variety of statistics on surfaces, including classical roughness values, peak and summit (texture) data and surface area calculations. The arithmetic average roughness R_a_ is calculated. The average roughness profile contains n ordered, equally spaced points along the trace. Height is assumed to be positive in the up direction, from the bulk material. The topographical features of the film coatings were also revealed by the AFM imaging as shown in Fig. 1. The average surface roughness of the Cu_2_O/CuO phase films prepared under the different deposition conditions is shown in Table 2.

**Table 2:** Average roughness of films at different deposition power ranges obtained using Atomic Force Microscopy (AFM).

Power (W)	Power density (W/cm^2^)	Predominant oxide phase (XRD)	Average roughness (nm)
200	0.6	Cu_2_O	2.1
300	0.9	Cu_2_O	3.9
400	1.2	Cu_2_O	2.6
500	1.5	Cu_2_O	4.6
600	1.9	Cu_2_O–CuO	10.2

Fourier transform infra-red spectroscopy on the films prepared on silicon wafer substrates was performed on a Nicolet Avatar360 spectrometer. XPS measurement was performed using a Scientia ESCA300-3U spectrometer with a monochromatic Al Kα (1486.6 eV) X-ray source on films prepared on microscope glass substrates. Reflectance ellipsometry measurement was performed on a Horiba Jobin Yvon iHR 320 ellipsometer. The acquisition routine was set at 190–850 nm 5 nm step 70-degree angle for the measurements of Psi (Ψ) and delta (Δ). The acquisition was performed on samples prepared on microscope glass and silica substrates at different conditions. The model we used consisted of a stack of layers consisting of the bulk substrate whose optical and dielectric constants were available in the literature, the thin film was modelled with a Tauc-Lorentz relationship and a rough top layer consisting of a 50% mixture of thin film and 50% void was included in the stack. This rough top surface was used in line with the Bruggeman effective medium approximation (BEMA). The measured results were modelled using the Lorentzian model to determine the film thicknesses, optical constants (n and k), as well as the dielectric constants ε_1_ and ε_2_. We already had estimates of the surface roughness from our AFM measurements. Contact angle and surface energy measurements were performed on a CAM200 goniometer located using a single syringe for each of the three investigating liquids. The liquids are water, ethylene glycol, and diiodomethane. The software in the instrument uses the contact angle results to evaluate the surface energies. The measurements were performed on films prepared on microscope glass substrates. The XPS measurement was carried out at the national centre for electron spectroscopy and surface analysis (NCESS) laboratory in Daresbury, Warrington, U.K.

## Results and discussions

### FT-IR results

The FT-IR characterisation of films prepared at 400 W, 500 W, and 600 W and different oxygen flow rates and deposition pressures on Silicon wafer show single absorption band characteristics of the Cu(I)–O vibration in Cu_2_O which has been previously reported by the current authors^14^.

### XPS results

A summary of the binding energies and FWHM of deconvoluted Cu 2*p*_3/2_ and O 1*s* main peaks of the films investigated is shown in Table 3. The two peaks are positioned at 932.5 eV corresponding to Cu_2_O, and 933.6 eV corresponding to CuO with a FWHM of 1.1 and 2.9 eV respectively^14^.

**Table 3:** Extract of XPS binding energies (BE) and full width at half-maximum (β) of oxide films in the de-convoluted Cu 2*p*_3/2_ and O 1*s* core level regions.

RF power (W)	Power density (W/cm^2^)	O_2_ Flow rate (sccm)	Pressure (mTorr)	Binding energy (BE)/FWHM (β)							
				Cu 2*p*_3/2_				O 1*s*			
				BE (1)	β	BE (2)	β	BE (main)	β	BE satellite)	β
200	0.6	2	6	932.4	1.1	933.6	3.1	529.6	0.9	531.1	1.6
300	0.9	2	6	932.5	1.6	934.4	1.5	529.7	1.3	531.5	1.5
400	1.2	4	6	932.5	1.1	933.6	2.9	529.5	0.8	531.1	1.6
500	1.5	6	4	932.2	1.1	933.7	2.8	529.6	1.2	531.1	1.5
600	1.9	6	6	932.5	1.5	933.6	2.9	529.4	0.9	531.2	1.4

### Ellipsometry results

In the reflectance ellipsometry measurements, the oxide films on glass substrates were modelled using Tauc–Lorentz model to determine the film thickness and optical constants.

A tabulation of the optical constants at different deposition conditions as prepared and annealed at different temperatures are shown in Tables 4 and 5. The real and imaginary parts of the dielectric responses were evaluated using Eqs. (13–15). The evaluated real part of the dielectric constant ranges between 8.23–8.94 at 400 nm, and 6.41–7.89 at 800 nm for the as-prepared films and has an average value of 7.59–8.37 at 400 nm and 7.37–8.10 at 800 nm for the annealed films. The value of the real part of the dielectric constant is of the order of the measured value 9.8, and quoted values of 8.58, 10.26, and 7.5 for the bulk reported in the literature by other authors^25–27^.

**Table 4:** Tabulated optical constants in the visible region at different deposition conditions as prepared at 400 nm.

Power (W)	O_2_ flow (sccm)	Press (mTorr)	As prepared at 400 nm			
			n	k	ε_1_	ε_2_
200	2	3.0	3.02	0.75	8.53	4.52
300	3	6.0	2.70	0.77	8.23	4.57
400	4	6.0	3.18	1.09	8.94	6.93
500	6	4.0	3.13	0.98	8.86	6.15
600	6	6.0	3.13	1.01	8.77	6.31

**Table 5:** Tabulated optical constants in the visible region at different deposition conditions as prepared at 800 nm.

Power (W)	O_2_ flow (sccm)	Pressure (mTorr)	As prepared at 800 nm			
			n	k	ε_1_	ε_2_
200	2	3.0	2.74	0.14	7.52	0.74
300	3	6.0	2.53	0.003	6.41	0.02
400	4	6.0	2.78	0.00	7.74	0.00
500	6	4.0	2.73	0.09	7.43	0.51
600	6	6.0	2.81	0.00	7.89	0.00

**Figure 2:**
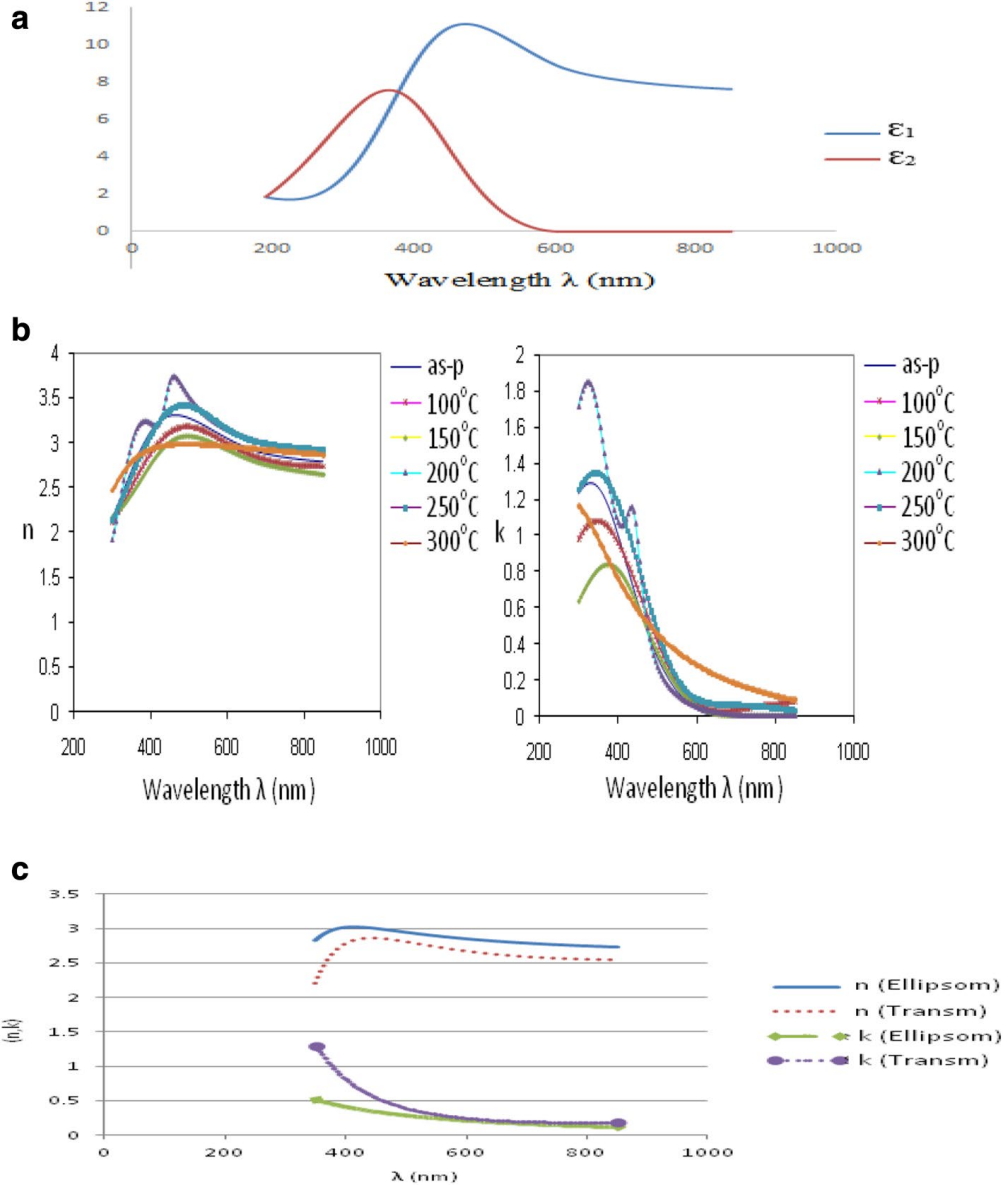
A typical plot of the dielectric constants real (ε_1_) and imaginary (ε_2_) parts obtained from reflectance ellipsometry measurement between 190 and 850 nm for the film prepared at 400 W power, 4 sccm oxygen flow rate and 6.0 mTorr pressure. (**b**) Optical constants (n and k) of film as-prepared on glass and silica (200 °C to 300 °C) substrates at the deposition conditions of 600 W-6sccm-6mTorr and annealed at different temperatures. (**c**) Typical plot for comparison of n and k from ellipsometry and optical transmission measurements for the film prepared on glass substrate at 200 W power (0.6 W/cm^2^), 2sccm oxygen flow and 3.0mTorr pressure.

A typical plot of the dielectric constants real (ε_1_) and imaginary (ε_2_) parts obtained from reflectance ellipsometry measurement between 190 and 850 nm for the film prepared at 400 W power, 4 sccm oxygen flow rate and 6.0 mTorr pressure is shown in Fig. 2a. A plot of the optical constants (n and k) of the as-prepared films is shown in Fig. 2b. The wavelength dependence of the refractive index n of the oxide films agrees with reports in the literature^25,26^. The wavelength dependence of the extinction coefficient k of the films also agrees with earlier reports in the literature^25,26^. The spectral response of the optical constants (n and k) shows little variation with changes in temperature. The band gap was evaluated from results of optical transmission measurements using nkd-8000 Aquila spectrometer^14^. The optical constants (n and k) obtained from ellipsometry and optical transmission measurements were compared for some samples to confirm the reliability of the data as shown in Fig. 2c. The thicknesses of these films obtained from the spectrophotometry and ellipsometry data was also compared as shown in the Table 6 with reasonable agreement between the results from both methods.

**Table 6:** A comparison of thicknesses obtained from ellipsometry and optical transmission measurements.

Power (W)	Power density (W/cm^2^)	Pressure (mTorr)	Oxygen flow (sccm)	Thickness (nm) [ellipsometry]	Thickness (nm) [transmission]
200	0.6	3.0	2.0	73.43	73.55
300	0.9	4.0	4.0	102.07	101.23
400	1.2	4.0	6.0	146.21	140.27
500	1.5	4.0	6.0	256.03	268.38
600	1.9	3.0	6.0	228.42	228.89

### Contact angle-surface energy results

Tables 7 shows the surface energy terms of films prepared at different rf power and oxygen flow rates. The range of surface energy components in this report is within reported values by Goto et al.^28^ for single phase CuO and Ogwu et al.^11^ for predominant Cu_2_O and Cu_2_O–CuO phases.

**Table 7:** The Fowlkes, Wu, and AB Surface Energy terms in mJ/m^2^ for Copper Oxide films compared at different rf Power and 4 sccm oxygen flow.

RF power (W)	O_2_ flow (sccm)	Fowkes γ^total^	Fowkes γ^d^	Fowkes γ^p^	Wu γ^total^	Wu γ^d^	Wu γ^p^	AB γ^total^	AB γ^lw^	AB γ^+^	AB γ^-^
200	4	38.13	33.79	4.34	43.07	35.90	7.17	37.00	37.83	0.13	3.12
300	4	41.16	36.82	4.34	36.88	39.97	6.90	37.91	43.66	0.73	3.77
400	4	25.21	23.39	1.82	29.73	26.12	3.61	25.29	24.39	0.27	1.65
500	4	31.33	31.32	0.01	31.87	31.98	− 0.10	29.87	30.13	0.30	0.43
600	4	29.92	29.90	0.02	32.03	32.08	− 0.05	30.60	30.74	0.17	0.39

### Evaluation of Hamakers constants

**Figure 3:**
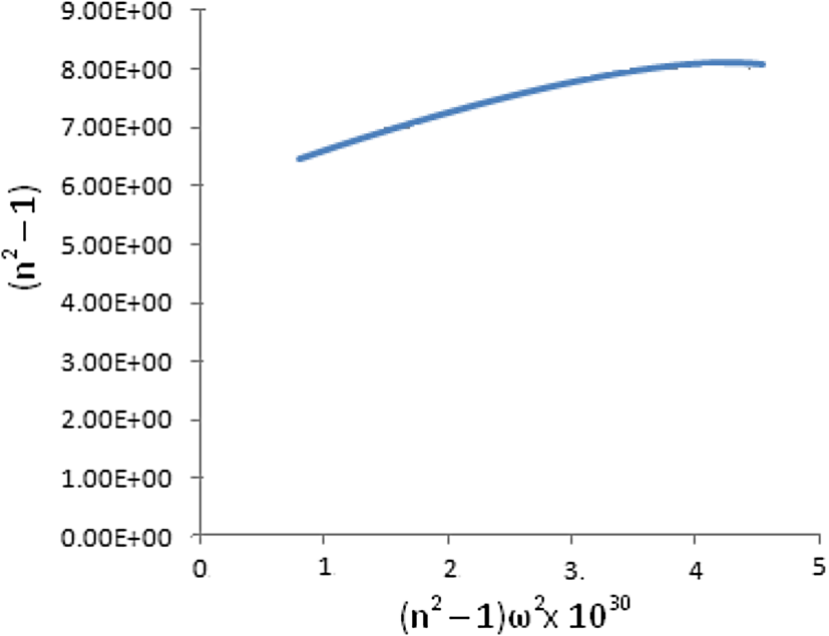
Cauchy plot of Cu_2_O/CuO material prepared at 200 W rf power.

The Cauchy plot was performed using Eq. 20 as shown typically in Fig. 3 for the film prepared at 200 W rf power to evaluate the function oscillator strength C_UV_ and frequency ω_UV_ in the ultraviolet region**.** The function oscillator strength in the infrared region was evaluated from Eq. 19 using C_UV_ and the static permittivity from Eq. 18.The Tarbo–Winterton (T–W) approximation evaluation was performed with v_e_ = 3 × 10^15^ s^-1^and T = 298 K in Eq. 21 from the Ellipsometry results to obtain the Hamaker constant for Cu_2_O/CuO|Water|/Cu_2_O/CuO and Cu_2_O/CuO|Air|/Cu_2_O/CuO structure for film materials prepared at different deposition rf power. The surface energy (dispersive component of Fowkes contribution) results were also used in Eq. 22 to evaluate the Hamaker constants. The dielectric constants from ellipsometry measurement were also used in Eq. 23 in the evaluation of the Hamaker constants. A comparison of these evaluations is given in Table 8.

**Table 8:** Table of non retarded Hamaker constants evaluated from ellipsometry and surface energy results.

RF power (W)	Material1	Medium	Material2	Hamakers constant (J) from Ellipsometry measurement (T–W) method	Hamakers constant (J) On the basis of Liftshitz theory using sampling frequency ^21^	Hamakers constant (J) from surface energy (XDLVU)	C_UV_	C_IR_
200	Cu_2_O/CuO	Water	Cu_2_O/CuO	3.45 × 10^–19^	1.25 × 10^–19^	0.94 × 10^–19^	6.14	3.57
	Cu_2_O/CuO	Air	Cu_2_O/CuO	4.88 × 10^–19^	3.40 × 10^–19^			
300	Cu_2_O/CuO	Water	Cu_2_O/CuO	3.02 × 10^–19^	3.90 × 10^–19^	0.71 × 10^–19^	4.93	4.78
	Cu_2_O/CuO	Air	Cu_2_O/CuO	4.43 × 10^–19^	3.22 × 10^–19^			
400	Cu_2_O/CuO	Water	Cu_2_O/CuO	3.98 × 10^–19^	3.57 × 10^–19^	0.49 × 10^–19^	6.03	3.68
	Cu_2_O/CuO	Air	Cu_2_O/CuO	5.41 × 10^–19^	3.58 × 10^–19^			
500	Cu_2_O/CuO	Water	Cu_2_O/Cu	3.62 × 10^–19^	3.70 × 10^–19^	0.62 × 10^–19^	5.96	3.75
	Cu_2_O/CuO	Air	Cu_2_O/CuO	5.05 × 10^–19^	3.46 × 10^–19^			
600	Cu_2_O/CuO	Water	Cu_2_O/CuO	3.99 × 10^–19^	3.57 × 10^–19^	0.51 × 10^–19^	6.34	3.41
	Cu_2_O/CuO	Air	Cu_2_O/CuO	5.42 × 10^–19^	3.59 × 10^–19^			

As shown in Table 8, there is a much better agreement in the range of Hamaker constant values with the limited Lifshitz approach and the Tabor-Winterton approximation (TWA), both of which involve data collected by ellipsometry, compared to those obtained through surface energy measurements. The surface roughness has a more pronounced effect on the surface energy, and this can be one of the contributors to the difference in Hamaker constant values using the surface energy measurements.

In addition, there are modifications to the contact angle/surface energy values which are approximated and not fully accounted for in the experimental measurements that include the effects of surface roughness, which is accounted for through the Cassie–Baxter and Wenzel equations as discussed earlier. A rigorous explanation of this effect has been shown through a variational formulation of the roughness dependence of surface free energy which has been already reported in the literature. Bormashenko^20^ developed a variational form for the energy of wetting on a surface given by the expression below,

24 $$ G = \mathop \int \limits_{0}^{a} \left[ {\gamma 2\pi x \sqrt {1 + \left( {\frac{dh}{{dx}}} \right)^{2} + - - - } } \right]dx $$

where h (x, t} is the local height at position x of a liquid surface above a substrate and γ is the surface tension. The role of geometry through the roughness on the fluid deformation, surface tension and van der Waals/Casmir force interaction is also captured within the augmented Young–Laplace equation^29^ expressed as,

25 $$ \gamma \nabla . \left( {\frac{\nabla \psi }{{\sqrt {1 + \left| {\nabla \psi } \right|^{2} } }}} \right) + \frac{\delta }{\delta \psi } \left( {\varepsilon_{other} \left[ \psi \right] + \varepsilon_{vdw} \left[ \psi \right]} \right) = 0 $$

Which describes the local balance of forces (Variational derivatives of energy) acting on a fluid with surface profile ψ(x), γ is the surface tension, the first two terms denote surface, gravity and other forces and the third term represents the van der Waals interaction energy.

## Conclusion

We have measured the Hamaker constant of copper oxide thin films using experimental optical and surface energy data. Copper oxide films belong to the group of anti-stiction coatings that hold substantial promise for applications in micro-electromechanical (MEMS) devices and with shrinking dimensions on the nanometre scale and nano electromechanical (NEMS) devices. We observed a much better agreement in the range of Hamaker constant values obtained between the limited Lifshitz approach and the Tabor–Winterton approximation (TWA), compared to those obtained through surface energy measurements. The Hamaker constants evaluated based on surface energy have much lower values. This difference is to be expected since the surface energy was evaluated from contributions due to the three liquids used i.e. water, ethylene glycol, and diiodomethane. The use of three liquids namely water, ethylene glycol and diiodomethane also requires the use of combining rules such as the geometric mean approximation for evaluating the surface energies as earlier stated in this paper, that will affect the values of Hamaker constant calculated from the surface energy measurements. However, it is our opinion that the three methods adopted in this paper, have substantial advantages over the use of the Atomic force microscope (AFM) to determine the Hamaker constant. The challenges with using the AFM to determine the Hamaker constant include, cantilever tip-surface interaction, surface deformation, van der Waals induced jump contact, cantilever mechanics and velocity, all of which can introduce uncertainties in the determination of the Hamaker constant using the AFM. The approximate Lifshitz theory using ellipsometry data is less prone to uncertainties in measurements compared to the other two methods we used and to the AFM approach used by others in the literature. It will be a more suitable technique for measuring the Hamaker constant on the surface of anti-stiction coatings to be used on MEMS, NEMS and other nano-systems where stiction will pose a performance challenge now and in the future.

## References

[1] Fathipour S, Almeider SF, Ye ZA, Saha B, Nirai F, Liu T-K, Wu J (2019). Reducing adhesion energy of nano-electromechanical relay contacts by self-assembled perfluoro (2,3-dimethylbutan-2-1), coating. AIP Adv..

[2] Rahmanian S, Hosseini-Hasheimi S, SoltanRezaee M (2019). Efficient large amplitude primary resonance in extensional nanocapacitors: nonlinear mean curvature component. Sci. Rep..

[3] Xu L, Liu Y, Fu X (2016). Effects of the Van der Waals force on the dynamic performance of a micro-resonant pressure sensor. J. Shock Vib..

[4] Soon BW, Ng EJ, Qian Y, Singh N, Tsai MJ (2013). A bi-stable Nano electro-mechanical non –volatile memory based on Van der Waals force. Appl. Phys. Lett..

[5] Soroush R, Koochi A, Kazemi AS, Noghrehabadi A (2010). Investigating the effect of Casmir and Van der Waals attractions on the electrostatic pull-in stability of nano-actuators. Phys. Scr..

[6] Batra RC, Porfini M, Spinello D (2007). Effects of Casmir force. Eur. Phys. Lett..

[7] Palasantzas G, De Hosson J (2005). Pull-in characteristics of electromechanical switches in the presence of Casmir forces: Influence of self-affine surface roughness. Phys. Rev. B.

[8] Palasantzas G, Van Zwol PJ, De Hosson J (2008). Transitions from Casmir to Van der Waals force between microscopic bodies. Appl. Phys. Lett..

[9] Van Zwol PJ, Palasantzas G, De Hosson J (2008). Influence of random roughness on the Casmir force at small separations. Phys. Rev. B.

[10] Eizner E, Horovitz B, Hemkel C (2012). Waals-Casmir-Poldar interaction of an atom with a composite surface. Eur. Phys. J. D..

[11] Ogwu AA, Bouquerel E, Ademosu O, Moh S, Crossan E, Placido F (2005). An extended Derjaguin–Landau–Verwey–Overbeek theory approach to determining the surface energy of copper oxide thin films prepared by reactive magnetron sputtering. Metall. Mater. Trans. A.

[12] Lim T-C (2003). The relationship between Lennard-Jones (12–6) and Morse potential functions. Z. Naturforsch..

[13] Morley SD, Abraham RJ, Haworth IS, Vinter JG (1991). Cosmic (90): An improved molecular mechanics treatment of hydrocarbons and conjugated systems. J. Computer-aided Mol. Des..

[14] Ogwu AA, Darma TH (2013). A reactive magnetron sputtering route for attaining a controlled core-rim partitioning of/CuO thin films with resistive switching potential. J. Appl. Phys..

[15] Edwards SF, Wilkinson DR (1982). The surface statistics of a granular aggregate. Proc. R. Soc. Lond.

[16] Kardar M, Parisi G, Zhang Y-C (1986). Dynamic scaling of growing interfaces. Phys. Rev. Lett..

[17] Watson SMD, Houlton A, Horrocks B (2012). Equilibrium and non-equilibrium thermodynamics of templating reactions for the formation of nanowires. Nanotechnology.

[18] Barato AC (2010). Non-equilibrium wetting. J. Stat. Phys.

[19] Lipowsky R (2010). Non-linear growth of wetting layers. J. Phys A.

[20] Bormashenko E (2011). General equation describing wetting of rough surfaces. J. Colloid Interface Sci..

[21] Bergstrom L (1997). Hamaker constants of inorganic materials. Adv. Coll. Interface. Sci..

[22] Masuda T, Matsuki Y, Shimoda T (2009). Spectral parameters and Hamaker constants of silicon hydride compounds and organic solvents. J. Colloid Interface Sci..

[23] Bergstrom L, Stemme S, Dahlfords T, Arwin H, Odberg L (1999). Spectroscopic ellipsometry characterisation and estimation of the Hamaker constant of cellulose. Cellulose.

[24] Lefevre, G. & Jolivet, A. Proceedings of International Conference on Heat Exchanger, Schladming, Austria, *Fouling and Cleaning* VIII June 14–19 (2009)

[25] Derin H, Kantarli K (2002). Optical characterisation of thin thermal oxide films on copper by ellipsometry. Appl. Phys. A.

[26] Drobny VF, Pulfrey DL (1979). Properties of reactively sputtered copper oxide thin films. Thin Solid Films.

[27] Yang W-Y, Kim W-G, Rhee S-W (2008). Radio-frequency sputter deposition of single phase cuprous oxide using Cu20 as a target material and its resistive switching properties. Thin Solid Films.

[28] Goto M, Kasahara A, Oishi T, Konishi Y, Tosa M (2003). Lubricative coatings of copper oxide for aerospace applications. J. Appl. Phys..

[29] Venkataram PS, Whitton JD, Rodriguez AW (2016). Non-additivity of van der Waals forces on liquid surfaces. Phys. Rev. E.

